# EBNA3C interacts with Gadd34 and counteracts the unfolded protein response

**DOI:** 10.1186/1743-422X-6-231

**Published:** 2009-12-29

**Authors:** Jose L Garrido, Seijii Maruo, Kenzo Takada, Adam Rosendorff

**Affiliations:** 1 University of Pittsburgh and Children's Hospital of Pittsburgh, One Children's Hospital Drive, 4401 Penn Ave, Pittsburgh, PA 15224, USA; 2 Department of Tumor Virology, Institute for Genetic Medicine, Hokkaido University, N15 W7, Kita-ku, Sapporo 060-0815, Japan

## Abstract

EBNA3C is an EBV-encoded nuclear protein, essential for proliferation of EBV infected B-lymphocytes. Using EBNA3C amino acids 365-545 in a yeast two hybrid screen, we found an interaction with the Growth Arrest and DNA-damage protein, Gadd34. When both proteins are overexpressed, Gadd34 can interact with EBNA3C in both nuclear and cytoplasmic compartments. Amino acids 483-610 of Gadd34, including the two PP1a interaction, and the HSV-1 ICPγ34.5 homology domains, are required for the interaction. Furthermore, interaction is lost with a mutant of EBNA3C (^509 ^DVIEVID ^515^→AVIAVIA), that abolishes EBNA3C coactivation ability as well as SUMO interaction[1]. In B-cells, Gadd34, and EBNA3C are present in a complex with PP1a using microcystin sepharose affinity purification, Using a lymphoblastoid cell line in which EBNA3C protein levels are conditional on hydroxytamoxifen, surprisingly, we found that (i) EBNA3C *maintains *phosphorylation of eIF2α at serine 51, and (ii) protects against ER stress induced activation of the unfolded protein response as measured by XBP1 (u) versus XBP1(s) protein expression and N-terminal ATF6 cleavage. In reporter assays, overexpression of Gadd34 enhances EBNA3C's ability to co-activate EBNA2 activation of the LMP1 promoter. Collectively the data suggest that EBNA3C interacts with Gadd34, activating the upstream component of the UPR (eIF2α phosphorylation) while preventing downstream UPR events (XBP1 activation and ATF6 cleavage).

## Background

Epstein-Barr virus is a ubiquitous human herpes-virus that causes infectious mononucleosis. It remains latent in B-cells following resolution of infection, however, it has the potential to be a serious opportunistic pathogen. Expression of EBV latency III proteins is observed in acute infection, as well as in EBV positive post-transplant, and X-linked lymphoproliferative disease (PTLD and XLP) and HIV associated CNS lymphoma[2]. In this pattern of gene expression, 6 nuclear proteins (EBNAs 1,2 3A, 3B and 3C), three integral membrane proteins (LMP1, LMP2a and LMP2b) and two non-coding poly-adenylated RNAs (EBERS 1 and 2) are expressed[3,4]. Expression of these genes converts B-cells to leukemic lymphoblasts in vivo, and to lymphoblastoid cell lines in vitro. EBNA3C, is essential for initiation of B-cell growth, as well as ongoing B-cell transformation. Recombinant EBV containing a stop codon in the EBNA3C ORF is able to cause B-cell transformation only when transcomplemented for wild-type EBNA3C either in cis or trans, and LCLs immortalized by recombinant EBV containing a conditional EBNA3C gene, undergo growth arrest when EBNA3C expression is turned off [5-7]

EBNA3C co-activates transcription with EBNA2 at the viral LMP-1 promoter, as well as heterologous reporter systems designed to test p300 function. EBNA3C amino acids 343-545 were found to be essential for co-activation in both reporter systems, and yeast two-hybrid studies established that aa365-545 are sufficient for interaction with both SUMO-1 and with SUMO-3 [8] We further established that EBNA3C uses a SUMO interaction motif (SIM) (aa 507-513) to interact with SUMO-1 and SUMO-3, and that co-activation with EBNA2, is lost with mutations of the SIM (eg. m2, ^509 ^DVIEVID ^515^→AVIAVIA) that prevent SUMO binding, as well as with larger deletions (eg Δ343-545) that remove the central portion of the protein including the SIM, but leave other structural domains (eg the RBP-J-Kappa binding domain) intact [1]. In an effort to define other transcriptional activators associated with EBNA3C in SIM dependant manner, we performed a yeast two hybrid assay using EBNA3C aa365-545 as bait, and a splenic B-cell yeast two hybrid library as prey.

Suprisingly, EBNA3C was shown to interact robustly with the Growth Arrest and DNA-damage protein 34 (Gadd34), an ER-associated protein that is up-regulated in response to viral infection as well as ER-stress. Furthermore, interaction with Gadd34 was lost when we tested a SIM mutated form of EBNA3C for interaction (m2, ^509 ^DVIEVID ^515^→AVIAVIA)

In this study we sought understand the effects of the interaction between EBNA3C and Gadd34 on transcriptional co-activation with EBNA2 at, the -512/+72 LMP-1 promoter. Since Gadd34 is involved in resuming protein synthesis following resolution of ER stress, by functioning as a phosphotase subunit towards eIF2α, we also sought to investigate EBNA3C effects on the unfolded protein response in EBV infected B-lymphocytes.

In this study, we map a region important for EBNA3C interaction with Gadd34, and show that Gadd34 can cooperate with EBNA3C in co-activation of the LMP1 promoter with EBNA2, in a manner that depends on EBNA3C interaction with Gadd34, but appears independent from Gadd34 binding to PP1a. Using a cell line (LCL C19-9) conditional on Tamoxifen for EBNA3C expression, surprisingly, we find that EBNA3C expression results in an increase in eIF2α serine 51 phosphorylation, an early event in both the PKR and unfolded protein responses. Paradoxically, EBNA3C protected against downstream events in the UPR, namely the switch from expression of unspliced to spliced XBP1 isoforms, as well as ATF6 cleavage. EBNA3C's interaction with Gadd34 may therefore sustain LMP1 promoter activation in latency III infection, while preventing stress-induced activation of the UPR.

## Materials and methods

### Plasmids

Plasmids psg5-EBNA2, psg-5 EBNA3C 11-992, psg-5 EBNA3C 11-992 ^509^DVIEVID^515^→^509^AVIAVIA^515(^M2) and reporter plasmids -512/+72 LMP-1-Luc, and pgkB-Gal, have been described previously. Plasmids pAS EBNA3C aa365-545 was constructed by subcloning the BamHI/SalI fragment from pGEX-3X-EBNA3C aa365-545 into the BamHI and SalI sites of pAS-1 (Gift of S. Elledge). pAS EBNA3C aa365-545-M2 was constructed by subcloning the SpeI/AatII fragment from psg-5 EBNA3C-M2 into pAS-1 EBNA3C aa365-545. psg5-Flag-Gadd34 1-674, 180-674, 180-610 and 180-483 were kindly provided by M Brush and S Shenolikar, Duke University.

### Antibodies

Anti-human Gadd34(ab9869), anti-XBP1 (ab37152), anti-Laminin B (ab16048), and anti-eIF2α (ab5369) antibodies were purchased from Abcam Inc. Anti-phospho-eIF2α (Ser51) antibody was obtained from Cell Signaling Technology. Anti-EBNA3C was purchased from Exalpha Biologicals Inc. Anitbody recognizing only the spliced XBP1 isoform was obtained from Biolegend. Anti-ATF6 antibody, recognizing only the 90 KDa isoform, was purched from Imgenex (San-Diego, CA). Anti-β-actin (A1978) antibody was obtained from Sigma-Aldrich.

#### Yeast two hybrid analysis

50 μg of pAS-1 EBNA3C aa365-545 and a pACT library were co-transformed into 1.5 mL of log phase yeast strain AH109. Yeast were spread on 40 150 mM synthetic dropout (SD) plates (Leu^-^, Trp^-^, His^-^, Ade^-^,β-gal) After 2 weeks of growth, 88 blue colonies were selected and replated on SD plates (Leu^-^, Trp^-^, His^- ^+25 mM 3-AT). 43/88 colonies that grew under these conditions, were selected and transforming plasmids segregated by two cycles of liquid culture amplification and restreaking SD (Leu^-^, Trp^-^, X-β-gal plates), yielding a mixture of blue and white colonies. Blue colonies were picked and yeast minipreps performed, followed by transformation of DH5α MAX Efficiency cells (Life Technologies, Rockville, MD) and miniprep. Duplicate cDNAs were identified by PCR across the pACT MCS, followed by Alu digest of PCR products. Unique cDNAs were then retested by retransformation into AH109 cells and growth on SD media (Leu^-^, Trp^-^, His^-^, Ade^-^.) Confirmed interactors were selected for parallel retransformation of AH109 with EBNA3C 1-992, EBNA3C aa365-545 or EBNA3C aa365-545 M2, followed by plating on SD media (Leu^-^, Trp^-^, His^-^, Ade^-^).

### Microcystin pulldowns and Co-immunoprecipitation

4 million BJAB or BJAB-E3C cells were collected and lysed in 1 mL buffer containing 0.5% NP-140, 150 mM NaCl with or without added 0.5% BSA. Lysates were incubated for 1 hour or overnight with either protein G sepharose (Amersham Pharmacia, Piscataway, NJ) or microcystin sepharose (Upstate, Lake Placid, NY). Proteins were resolved by electrophoresis and EBNA3C and Gadd34 were detected using mouse monoclonal A10 antibody, and anti-Gadd34 rabbit polyclonal antibody (C-20, Santa Cruz) For IB4 co-immunoprecipitation experiments, 15 million IB4 cells were lysed in RIPA buffer (1% NP-40, 1% sodium deoxycholate, 0.1% SDS, 0.15 molar NaCl, 0.01 molar sodium phosphate, pH 7.2) for 30 minutes. Insoluble matrix was pelleted by centrifugation at 12 000 RPM for 10 minutes, and clarified lysates pre-cleared with protein-G-sepharose. Protein-G-Sepharose or Protein-G sepharose with A10 antibody were added to lysates which were incubated with rotation at 4 degrees overnight. Following IP, proteins were resolved by electrophoresis and Gadd34 detected using C-20 antibody.

### Reporter Assays

BJAB cells (10^7^) were transfected in 0.4 mL of RPMI 1640 supplemented with 10% Neugem serum with a Bio-Rad gene pulser at 200V and 960 μF. Each transfection contained 5 ug of -512/+72 LMP1-Luc plasmid and 5 ug pgk-B-Galactosidase plasmid as a normalization control, and the indicated microgram amounts of psg5-EBNA2, psg5-EBNA3C or psg5-Gadd34 plasmids. Total plasmid DNA was made equal across transfections by the addition of psg5 vector DNA. After transfection, cells were placed in 10 mL of complete medium and incubated at 37 for 24 hours. Cells were collected, washed in phosphate buffered saline, lysed in reporter buffer (Luciferase Assay System, Promega, Madison, WI) by one freeze thaw cycle, and assayed for luciferase and beta-galactosidase activities (Galacto-Light; Tropix) with an Opticomp I luminometer (MGM instruments.)

### Stress assays

LCL C19-9 cells were maintained in RPMI 1640 suplemented with 15% FBS in the presence of 400 nM - hydroxytamoxifen (4-HT) (Sigma-Aldrich), and then transferred to medium containing either 4 HT or DMSO for 9 days. Six hours prior to harvest, cells were treated with Thapsigargin (0.5 μM) to induce ER stress and activate the UPR. Following treatment cells lysed were prepared using RIPA buffer. Protein concentration was measured using Bio-Rad protein asssay. Equal amounts of protein (20 μg) were separated by SDS-PAGE and protein transferred to a PVDF membrane. Blots were blocked by incubation for 1 h at room temperature in 3% bovine serum albumin (BSA) in TBS buffer (10 mM Tris HCl pH 7.6; 150 mM) containing 0.1% Tween-20 (TBS-T). The membranes were incubated overnight with the indicated primary antibodies diluted in 1% BSA in TBS-T. Membranes then were washed 3 times for 10 min with TBS-T followed by incubation with the appropriate secondary antibodies diluted in 1% BSA in TBS-T for 1 h at room temperature. Finally, membranes were washed 5 times with TBS-T buffer for 15 min and immunoreactivity was detected using ECL system obtained from Millipore.

## Results and Discussion

### The EBNA3C SUMO interaction motif is required for interaction with Gadd34

Yeast two-hybrid screening using EBNA3C aa365-545 as bait, revealed one of the interactors to be the protein encoded by pACT-Gadd34 aa395-674. Interaction was confirmed with a β-galactosidase filter lift assay, after an half hour incubation. Previous studies showed that EBNA3C aa365-545 containing the m1 (507 DDDVIEVID 515→DDAVIAVIA) and m2 (507DDDVIEVID 515→AAAVIEVID) interact very weakly with SUMO-1 and fail to interact with SUMO-3[1]. To test whether interaction between EBNA3C and Gadd34 also depends on the SUMO interaction motif, the m2 mutation was constructed in the context of EBNA3C aa365-545, and tested against Gadd34 aa395-674 (Table 1). While wild type EBNA3C aa365-545 interacted with Gadd34, interaction was lost when the m2 mutant was tested. As expected, wild type EBNA3C aa365-545 but not the m2 mutant, interacted with SUMO-3 [1]. Therefore EBNA3C aa365-545 interaction with Gadd34 depends on the presence of an intact SUMO interaction motif, a domain of EBNA3C also required for transcriptional co-activation with EBNA2.

**Table 1:** Focused yeast two-hybrid assays

	1-992	aa365-545	aa365-545 m2
**SUMO-3**	++	+++	-

**RBP-J-κ**	+++	-	-

**Gadd34**	++	+++	-

Focused yeast two-hybrid assays testing the interaction of full-length EBNA3C, EBNA3C aa365-545 Yeast strain AH109 were transformed in parallel with pAS1-EBNA3C (1-992), pAS-WT EBNA3C (aa365-545) or with the SIM (M2) mutant (aa365-545, ^509^DVIEVID^515^→^509^AVIAVIA^515^) and tested for interaction with pACT-Gadd34aa395-674. Colonies were sampled using a filter lift assay and interaction confirmed using a LacZ assay. Results of a 1 hour LacZ assay, scored semi-quantitatively, are shown.

In order to test whether full length EBNA3C interacts with Gadd34, the Gal4 DNA binding domain was fused N-terminal to the entire EBNA3C ORF (aa1-992), cloned into Y2H vector pAS-1, and tested against pACT-Gadd34 aa395-674. As a positive control a cDNA encoding full length RBP-J-Kappa, cloned into pACT, was tested for interaction with EBNA3C under the same conditions. Full length EBNA3C interacted with Gadd34aa395-674, as well as with RBP-J-Kappa, and LacZ conversion occurred within 30 minutes for both interactions.

These data indicate that EBNA3C interacts with Gadd34aa395-674, and that the interaction is comparable in affinity to the interaction between EBNA3C and RBP-J-κ. Furthermore, EBNA3C aa365-545 interaction with Gadd34aa395-674 depends on the SUMO interaction motif. These data also suggest that Gadd34 may, in part, be a mediator of EBNA3C co-activation function.

### EBNA3C interaction with Gadd34 requires Gadd34 amino acids 483-610

**Figure 1:**
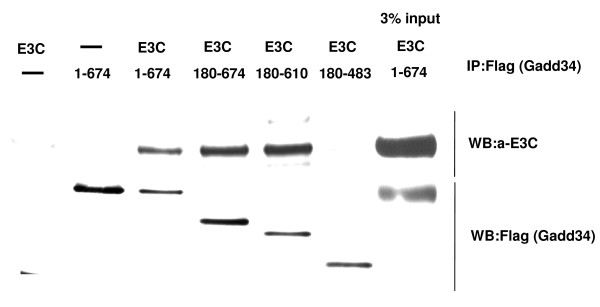
**Gadd34 aa 483-610 are required for EBNA3C association**. a) 293-T cells were seeded in 6-well plates resulting in 70% confluence by day 2, and transfected with 0.5 μg of either psg5-EBNA3C alone (lane 1), psg5-Flag-Gadd34 (full-length, aa1-674, lane 2), or both EBNA3C and Flag-Gadd34 (1-674) (lane 3). In lanes 4-7 a combination of EBNA3C and the indicated Gadd34 mutants were transfected. Total DNA was 1.0 μg for each transfection. 18 hours post-transfection, cells were lysed in isotonic buffer containing 0.5% NP-40. Gadd34 or Gadd34 mutant proteins were immune precipitated with anti-flag affinity beads (M2-agarose), and precipitating proteins western blotted with α-EBNA3C antibody (A10) (top panel) or Flag antibody (bottom panel). In lane 7, 3% of starting lysates prior to immune precipitation (input) from the well transfected with psg5-EBNA3C alone (top panel), or psg5-Flag-Gadd34 alone (bottom panel) is shown.

To test whether EBNA3C can interact with Gadd34 in human cells, and to better understand the domains of Gadd34 responsible for interaction with EBNA3C, 293T cells were transfected with expression vectors encoding either EBNA3C alone (figure 1, lane 1), flag-tagged full length Gadd34 alone (lane 2), both full length EBNA3C and Flag-tagged-Gadd34 (lane 3) or combinations of EBNA3C and the indicated Gadd34 constructs as indicated (lanes 4-6). Transfected Gadd34 was immunoprecipitated using sepharose beads coupled with anti-flag antibody (M2 beads), and interaction with EBNA3C tested by western blotting for both Gadd34 and EBNA3C. Both full length Gadd34(1-674), as well as an N-terminal truncation mutant (180-674) interacted with EBNA3C (lanes 3 and 4). Interaction was enhanced with further deletion of Gadd34 amino acids 611-674 (lane 5), however interaction was lost when Gadd34 aa180-483 was tested. This data indicates that Gadd34 interaction with EBNA3C requires Gadd34 amino acids 483-610. This domain includes the sequences 555-558 KVRF, and 612-616 RARA, which when mutated (KVRF→KARA) or deleted (ΔRARA), reduce activation and association with PP1a respectively. In lane 7, 3% of starting lysates prior to M2 flag pulldown is shown.

### EBNA3C interacts with Gadd34 and PP1a in human B cells

**Figure 2:**
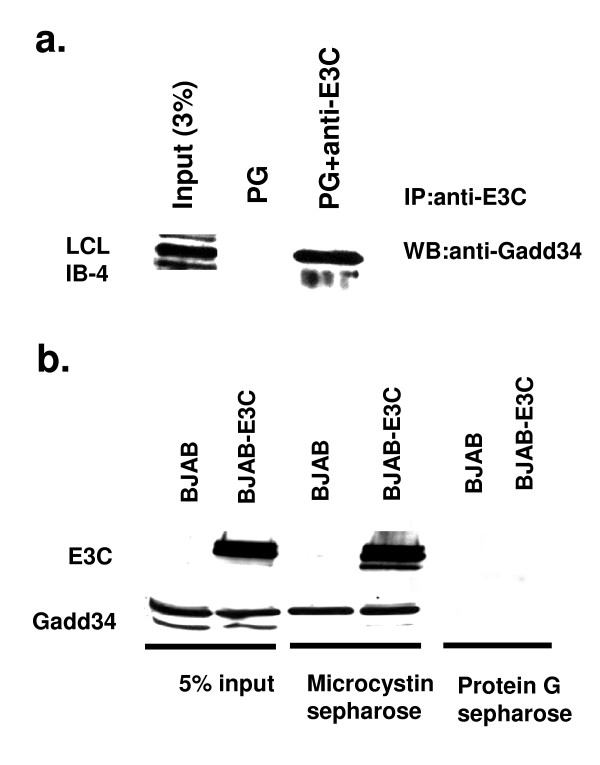
**EBNA3C interacts with Gadd34 by co-IP and co-purifies with Gadd34 by microcystin pulldown**. a) IB4 cells (5 million for each treatment) were collected, lysed in isotonic 0.5% NP-40 buffer, and immune precipitations (IP) performed with either protein G alone (PG) or protein G with the addition of anti-EBNA3C (A10) sera (PG/anti-EBNA3C) Proteins were western blotted for Gadd34 (SC-H193), following IP. b) Burkitt's lymphoma BJAB cells or BJAB-EBNA3C cells (5 million for each treatment) were collected, lysed in isotonic 0.5% NP-40 buffer, containing additional 0.5% BSA, and incubated with the indicated sepharose beads conjugated to either protein G or microcystin LR. Lysates and beads were rotated for 1 hr at 4 degrees, extensively washed with PBS, and affinity purified proteins western blotted for the presence of EBNA3C (E3C) and Gadd34.

To test whether EBNA3C interacts with Gadd34 in human B-cells, the lymphoblastoid cell line IB4, which contains integrated copies of the EBV B95-8 genome, was used. EBNA3C was immune precipitated from RIPA lysates, using protein G sepharose followed and A10 anti-EBNA3C antibody. Immunoblotting for Gadd34 revealed that EBNA3C associates with Gadd34 in IB4 LCLs (Figure 2a.)

In order to test whether EBNA3C resides in PP1a complexes in cells, microcystin sepharose was applied to RIPA buffer whole-cell lysates from BJAB cells which do not express EBNA3C or BJABs stably expressing EBNA3C (Figure 2b) Addition of protein G sepharose failed to immune precipitate EBNA3C from wild type BJABs or BJABs stably expressing EBNA3C (Figure 2b, lanes 5 and 6). Likewise microcystin sepharose failed to immune precipitate EBNA3C in wild-type BJABs (lane 3). However when microcystin sepharose was applied to BJABs stably expressing EBNA3C, a small amount, representing approximately 3-5% of input EBNA3C, co-precipitated (lane 4). In transient transfection experiments in 293T cells, EBNA3C was able to interact with Gadd34 in both cytoplasmic and nuclear compartments (data not shown).

Microcystins are cyclic heptapeptides synthesized by blue-green algae that covalently couple to the catalytic subunits of protein phosphotases PP1 and PP2a [9]. Although not completely specific to PP1a, taken together, the data indicate that both EBNA3C and Gadd34 reside in protein phosphotase complexes in B cells.

### Gadd34 overexpression enhances EBNA3C transcriptional co-activation with EBNA2

Since Gadd34 interaction with EBNA3C depends on a motif that is also required for transcriptional co-activation by EBNA3C reporter assays in the Burkitt's lymphoma cell line, BJAB were used to assess Gadd34 affects on EBNA3C co-activation. The reporter chosen was -512/+72 of the LMP-1 promoter fused to luciferase, which contains two RBP-J-Kappa binding sites, and is reliably activated by EBNA2 and further activated by EBNA3C [8]. This region of the LMP1 promoter also includes an ER stress response element (ERSE) located at position -146 relative to the transcriptional start site, recently shown to be important for LMP1 promoter activation by ER stress inducing chemicals (eg Brefeldin A, Tunicamycin) or by overexpression of the ER-stress transactivator, XBP1 [10].

**Figure 3:**
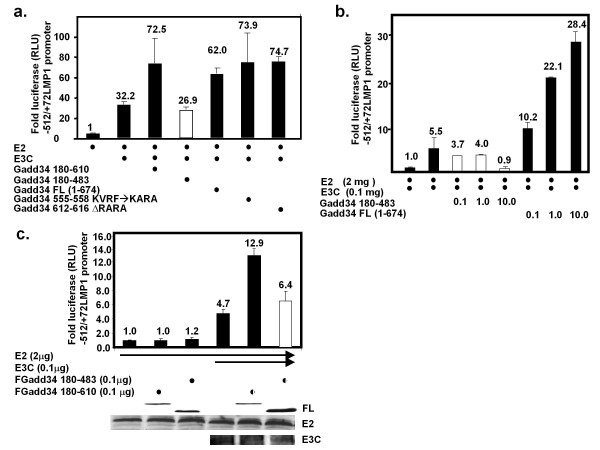
**A: Gadd34 co-activation with EBNA3C requires aa483-610, and is independent of PP1 recruitment**. A) BJAB cells (1 × 10^7^) were transfected with 5 μg of (-512/+72) LMP1p-Luc reporter construct, and 2 μg of psg5-EBNA2 (all lanes). psg5-EBNA3C (5 μg) was transfected(lanes 2-7). Gadd34 expression constructs were transfected as indicated (lanes 3-7) Luciferase values were normalized over beta-galactosidase levels obtained with co-transfection of 5 μg pgk-β-Gal plasmid. Reporter activation observed with EBNA2 alone is normalized to 1 (lane 1). Values are averages of duplicate observations in each experiment (repeated 3 times) plus standard error. A representative experiment is shown. **B: Gadd34 180-483 is dominant negative in a low-dose EBNA3C co-activation assay**. Reporter assay in BJAB cells with the indicated quantities of expression plasmids, as in figure 3a except that 100 ng (versus 5 μg) of psg5-EBNA3C expression plasmid was used. Each Gadd34 plasmids was titrated to determine stochiometry effects on EBNA3C, transfected at 0.1, 1.0 or 10.0 μg of DNA. **C: Gadd34 does not co-activate transcription with EBNA2 in the absence of EBNA3C **Reporter assay in BJAB cells with the indicated quantities of expression plasmids as in figure 3b. A representative experiment is shown. Protein expression levels of flag-tagged Gadd34, EBNA2 and EBNA3C are shown below.

LMP-1 promoter activity levels observed with expression of EBNA2 alone was normalized to 1, (Figure 3a, lane 1). Co-transfection of 10 μg EBNA3C expression plasmid increased reporter activity levels to over 32 fold that observed with EBNA2 alone (lane 2) while co-expression of EBNA3C *and *full length Gadd34 resulted in a further ~2.0 fold (from 32.2 to 62.0) increase in promoter activity levels (lane 5). Full length Gadd34 (aa 1-674, lane 5) and Gadd34 180-610 (lane 3) were equally effective in cooperating with EBNA3C, while full length Gadd34 had no effect on basal LMP1 reporter activity or EBNA2 activity in the absence of EBNA3C (data not shown). Gadd34 effects on LMP1 promoter activity depended on both EBNA2 and EBNA3C expression. In order to test whether the ability of Gadd34 to synergize with EBNA3C in the co-activation of EBNA2 at the LMP1 promoter, depended on Gadd34 binding to PP1a, point mutants known to affect PP1a activity (555KVRF558→KARA) or association (612-616ΔRARA) were tested in the same reporter assay (figure 3a). A 2-2.5 fold potentiation of EBNA3C promoter activity was again observed with both the KVRF (73.9 vs 72.5 for WT Gadd34) as well as ΔRARA (74.7 vs 72.5 for WT Gadd34) mutants (lane 2 versus lanes 5, 6 and 7). These data suggest that enhancement of EBNA3C activity by Gadd34 does not depend on association with PP1a.

When a smaller quantities (100 ng) of EBNA3C expression plasmid were used in the same experiment, EBNA3C expression increased LMP-1 promoter activity levels sub maximally to 5.5 fold relative to EBNA2 alone (Figure 3b, lane 2), and this was enhanced approximately 5-fold by the co-transfection of 10 μg of plasmid expressing Gadd34 (Figure 3b, lane 8). Gadd34 was able to potentiate EBNA3C transcriptional co-activation in a dose dependant manner (lanes 6-8). By contrast, a dominant negative effect on EBNA3C activity was observed with overexpression of a Gadd34 truncation mutant that cannot bind EBNA3C (Gadd34 aa180-483, figure 3b, lanes 3-5).

### EBNA3C expression is associated with eIF2a serine 51 phosphorylation

**Figure 4:**
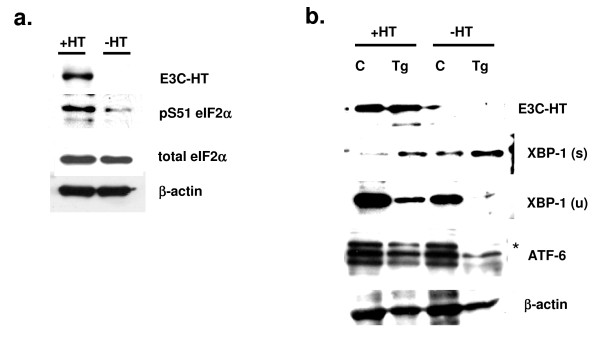
**A: EBNA3C effects on phosphorylation the translational control protein eIF2α**. LCL 19-9 was maintained in the presence (+HT) or absence (-HT) of hydroxytamoxifen for 10 days. Shown are western blots from whole-cell lysates against EBNA3C (top row), serine 51 phosphorylated eIF2α (second row), total eIF2α (third row) and β-actin loading control (fourth row). By day 10, in the absence of HT, LCL19-9 cells had stopped dividing [7]. **B: EBNA3C protects LCLs from activating the unfolded protein response**. As in Figure 4a except that cells were also treated with either DMSO "C" or 0.5 μM Thapsigargin "Tg", for the 4 hrs preceding harvest. XBP1(s) and XBP1(u) were individually detected using isoform specific antibodies. An ATF6 antibody that detects only full length, uncleaved ATF6 (90 KDa) was used (Imgenex, San Diego, CA).

EBNA3C interaction with Gadd34 might effect eIF2α phosphorylation, because Gadd34 recruits PP1a to the ER where the Gadd34/PP1a holoenzyme dephosphorylates eIF2a at serine 51. In order to determine EBNA3C effects on eIF2α activation, we used a cell line (LCL-19-9) in which EBNA3C expression is controlled by hydroxytamoxifen. Aliquots of these cells were maintained in media containing hydroxytamoxifen and then transferred to media with (+HT) or without (-HT) hydroxytamoxifen, for 8-10 days. Lysates were prepared and western blotted for the indicated proteins (Figure 4a) As expected with this cell line, withdrawal of hydroxytamoxifen resulted in slowed cell growth over a period of 8-10 days, accompanied by loss of EBNA3C-HT protein expression. While total eIF2α levels were comparable in cells grown in the presence of absence of tamoxifen, a significant difference in serine 51 phosphorylation was observed, with little effect on total eIF2α levels (Figure 4a).

### EBNA3C protects against thapsigargin induced endoplasmic reticulum stress

ER stress results in the endonucleolytic cleavage 26 nt of sequence from the XBP1 mRNA by the ER resident protease IRE1. The protein that is translated from the unspliced XBP1(u) mRNA, gives rise to a 33 Kda protein, XBP1 (u) with a functional DNA binding domain, but no activation domain. Following splicing by IRE1, a frameshift is introduced into the mRNA, resulting in the translation of a 54 Kda protein, XBP1(s) containing both DNA binding and activation domains. ATF6 is normally bound to the ER resident protein, Bip/GRP68. Upon accumulation of unfolded proteins in the ER, Bip dissociates from ATF6, and ATF6 translocates to the golgi where it is cleaved by site 1 and site 2 proteases, resulting in the disappearance of the full length 90 KDa protein, and the appearance of 50 KDa and other lower molecular weight cleavage products. XBP1(s) and cleaved ATF6 activate distinct and overlapping sets of proteins (such as chaperones and protein disulfide isomerases) important for resolution of ER stress during the UPR [11]. Thapsigargin induces ER stress by inactivating the ER Ca^2+ ^ATPase resulting in depletion of Ca^2+ ^from the luminal ER, and dysfunction of Ca dependant ER proteins such as calnexin.

In order to determine whether EBNA3C affects the UPR in LCLs, LCL C19-9 was maintained in the presence or absence of hydroxytamoxifen as in 4a. Four hours prior to harvest, cells were incubated in either DMSO alone (Figure 4b, "C") or in 0.5 μM Thapsigargin ("Tg"). Lysates from each treatment were blotted for the indicated proteins. All of the XBP1 protein was present in the unspliced form in control cells growing expressing EBNA3C-HT (Figure 4b, lane 1). Incubation with Thapsigargin resulted in a decrease in unspliced XBP1 protein (lane 2), and an increase in spliced XBP1. By contrast, in LCLs lacking EBNA3C-HT expression, XBP1(s) was detectable at baseline (lane 3), and XBP1(s) levels increased dramatically following the addition of thapsigargin (lane 4). Correspondingly, no XBP1(u) protein was detectable in LCLs exposed to Thapsigargin, in the absence of EBNA3C-HT. Similarly, while Thapsigargin resulted in a modest decrease in full-length, 90 KDa ATF6 in cells growing in the presence of EBNA3C-HT (band indicated by an asterisk, compare lane 1 to lane 2), in the absence of EBNA3C-HT, the 90 KDa form of ATF6 was undetectable (lane 4).

Collectively these data strongly implicate EBNA3C in preventing LCLs from undergoing UPR signaling, following ER stress.

## Conclusion

In this study, a yeast two-hybrid assay was conducted to reveal further EBNA3C interacting proteins. In particular our primary intention was to find novel proteins that might give clues as to the mechanism of EBNA3C transcriptional co-activation with EBNA2. Surprisingly, the two-hybrid assay failed to reveal convincing interactions with *bona-fide *nuclear transcription factors. Rather an interaction was discovered between EBNA3C and the translation control protein, Gadd34, and robustly confirmed in human cells by immune precipitation. Since mutations that affect SUMO-1 and SUMO-3 binding also affect the ability of EBNA3C to bind to Gadd34, as well as EBNA3C's ability to co-activate transcription with EBNA2, a genetic and biophysical link has been established between EBNA3C SUMO and Gadd34 binding and EBNA3C transcriptional activity. Further experiments clearly indicated that full length Gadd34, including aa 483-610 which were required for EBNA3C interaction, have a synergistic effect with EBNA3C in co-activating transcription with EBNA2 at the LMP1 promoter, while having no effect on EBNA2 driven reporter activity in the absence of EBNA3C. Indeed, a mutant of Gadd34, FL-Gadd34 aa180-483, acted as a dominant negative in reporter assays. It is known that the chromatin remodeling SWI-SNF protein, Ini-1 also associates with the C-terminus of Gadd34 explaining the positive effect on transcription. At higher concentrations (1 and 10 micrograms), Gadd34 180-483 functioned as a dominant negative, reversing EBNA3C coactivation. Since this mutant does not associate with EBNA3C, and is therefore likely not targeted to sites of EBNA3C activity on chromatin, the effect is likely due to swamping of the cell with Gadd34 protein, and sequestration of a positively acting factor such as the histone-acetyl transferase CBP, which is known to be required for full EBNA2 activity at the LMP1 promoter.

EBNA3C, a nuclear protein, would not be expected to interact with Gadd34, a cytosolic and ER-associated protein. However, EBNA3C might have the opportunity to associate transiently with Gadd34 at the ER following translation, during dismantling of the nuclear lamina during mitosis, or during lysosomal or proteasome or mediated degradation. Indeed EBNA3C and EBNA3A have been shown to associate with cytosolic proteasome subunits [12] Furthermore, while genetic studies have proven a role for EBNA3C in maintaining growth of EBV transformed B-lymphocytes, and EBNA3C biochemically fractionates to the nuclear compartment, it is unknown at present whether nuclear localization of EBNA3C *per se*, is required for EBV immortalization.

How might Gadd34 be important for EBNA3C transcriptional activity? One hypothesis, supported in the current study, is that EBNA3C negatively regulates Gadd34, decreasing the Gadd34-dependant PP1a recruitment to eIF2α, and consequently, increasing eIF2α phosphorylation. Since nuclear translocation of transcription factors involved in the PKR and UPR responses (such as ATF4, ATF6 and XBP1) usually occur downstream of eIF2α serine phosphorylation, a negative effect on Gadd34 function might be expected result in higher levels or greater activity of these transcription factors in the nucleus. Indeed there are numerous ATF4/XBP1 binding sites in the native LMP1 promoter as well as the -512/+72 construct used in this study, and expression of LMP1 has recently been shown to exert a feed forward effect by increasing eIF2α serine 51 phosphorylation, and downstream activation of ATF 4 [13]. At least two ER stress inducible elements have been described. These are controlled by ER stress induced transcription factors as well as XBP1. The first at position -41 relative to the transcriptional start site of the LMP1 gene, appears active in LCLs, is responsible for both EBNA2 dependant and EBNA2 independent activation of the LMP1 promoter in reporter assays, and is regulated by binding of ATF transcription factors [13-15]. The other ERSE (position -146) is active in NPC but not B-lymphocyte cell lines and is strictly required for LMP1 promoter activation by XBP1 overexpression or ER stress inducing chemicals such as Brefeldin A [10] Our initial hypothesis, therefore, was that EBNA3C potentiated UPR signaling, via an interaction with Gadd34, increasing ATF 4/6 and/or XBP activity, to co-activate LMP1 reporter activity with EBNA2.

Consistent with a negative effect on Gadd34 activity, EBNA3C expression was indeed associated with increased eIF2α serine 51 phosphorylation. Surprisingly, however, we found that EBNA3C expression was associated with *lower *levels of active XBP1(s), higher levels of inactive XBP(u), and increased levels of uncleaved (inactive) ATF6 protein in lymphoblastoid cells. Assuming that ATF and XBP transcription factors exert a positive effect on the LMP1 promoter in LCLs, Gadd34 is therefore unlikely to potentiate EBNA3C transcriptional effects by positively regulating ATF or XBP1 activity.

In our hands, EBNA3C's protective effects in LCLs appear similar to effects of the chemical Salubrinal on LCLs (data not shown), which has the effect of maintaining low levels of serine 51 phosphorylated eIF2α while preventing downstream events of the UPR such as the switch from XBP(u) to XBP1(s) protein expression, and activation by proteolytic cleavage of ATF6 in the Golgi. Salubrinal has been shown to protect neurons against ER-stress induced apoptosis, and EBNA3C may perform a similar role in LCLs[16]. Interestingly, Salubrinal also effects the efficiency of HSV-1 lytic replication, possibly through counteracting the effect of ICPγ34,5 on maintaining eIF2α dephosphorylation [17]. During the course of Coronavirus infection, eIF2a is also phosphorylated, resulting in a decrease in translation of host mRNAs, presumably favoring viral mRNA translation [18]. Although it is clear that EBNA3C expression is associated with eIF2α serine 51 phosphorylation, it is not clear why EBNA3C expression should result in eIF2α phosphorylation, a condition that would normally slow protein translation. Under conditions of eIF2a phosphorylation, the major alternative translational initiation factor eIF4 remains active, and translation is skewed towards 7-methylguanosine capped mRNAs, such as the mRNA for c-myc. Translation of viral rather than cellular RNAs may also be favored in this state. Further studies are needed to determine if EBNA3C is associated with a switch in translation from cellular to viral and oncogenic proteins.

Gadd34, PP1a and EBNA3C reside in a complex in Burkitt's lymphoma cells converted to permanent EBNA3C expression, using microcystin sepharose affinity purification of PP1 complexes. Overexpression of Gadd34 and EBNA3C in transient transfection experiments in 293T cells indicates that EBNA3C can interact with Gadd34 in both nuclear and cytoplasmic compartments. For instance, EBNA3C may delocalize Gadd34 to the nucleus, thereby decreasing the rate of PP1 recruitment, and consequently, increasing eIF2a phosphorylation.

Prolonged ER stress can result in apoptosis via ASK1 binding to IRE1, JNK activation, and phosphorylation of Bcl2 or CHOP mediated downregulation of Bcl2 levels[19] It therefore it seems plausible that EBNA3C is anti-apoptotic under conditions of prolonged ER stress such as might occur during initial EBV infection of resting B lymphocytes. XBP1, in concert with protein kinase D, can activate lytic EBV promoters such as the BZLF1 (Zta) promoter [20] Consistent with tight control of type III latency, we detect minimal XBP1(s) protein under normal LCL growth conditions, slightly increased XBP1 (s) protein upon EBNA3C withdrawal, and robust XBP1(s) expression under conditions of EBNA3C withdrawal in the face of chemical induced ER stress. We have also demonstrated that LCLs lytic promoters such as BZLF1, BMRF1 and BRLF1 are activated by Thapsigargin (manuscript in preparation). It seems likely, therefore, that one function of EBNA3C would be to maintain type III latency restriction, via shutoff of the UPR. Physiologically, EBV infected B-lymphocytes may be forced into lytic reactivation in the oropharyngeal mucosa, possibly undergoing ER stress secondary to BCR activation.

EBNA3C effects on transcriptional co-activation with EBNA2 may be separable from effects on the UPR, because the Gadd34 specific enhancement of EBNA3C transcriptional effects is maintained with overexpression of a Gadd34 mutant deficient in PP1a binding. However these results should be interpreted with caution. While the ΔRARA mutation eliminates PP1a binding, protein phosphotases are notoriously unstable in cells. Therefore apparent loss of PP1a interaction observed with this mutant, may in fact merely represent decreased association, and it is more likely that these mutations reduce PP1a phosphotase function and interaction rather than abolish it completely[21].

While viral infection activates PKR via dsRNA or interferon, PERK can be activated by ER stress. Both pathways of kinase activation occur during viral infection. Both PKR and PERK can phosphorylate eIF2α at serine 51, arresting cellular protein synthesis, as well as the synthesis of latent or lytic viral proteins. Gadd34 recruits PP1a to the endoplasmic reticulum where it dephosphorylates eIF2α, and is therefore essential in maintaining protein synthesis during viral infection[22,21] Herpesviruses have evolved mechanisms to counteract both PKR activation via Us11 expression, and eIF2α phosphorylation through ICPγ34.5 expression. Specifically, the HSV-1 encoded ICPγ34.5 protein accelerates and maintains the dephosphorylated state of eIF2α to ensure ongoing translation of viral mRNAs [23]. In this study, EBNA3C expression had the opposite effect, increasing rather than decreasing eIF2a phosphorylation, while paradoxically preventing the activation of downstream UPR. EBNA3C may therefore use the UPR pathway to alter the levels, activity, or network association, of numerous transcription factors in infected B cells, thereby modulating transcription of essential viral (eg LMP1) and cellular genes, while preventing UPR signaling, lytic reactivation, and downstream ER stress induced apoptosis.

These data presented in this study provide motivation for further studies of the effects of EBNA3C and other latency III proteins on the unfolded protein and PKR responses in EBV infection.

## Competing interests

The authors declare that they have no competing interests.

## Authors' contributions

AR provided the scientific hypotheses, performed experiments shown in table 1, and figures 1, 2, 3, and wrote the paper. JLG performed experiments shown in figure 4. SM made and provided the EBV immortalized B-cell line LCL 19-9. All authors read and approved the final manuscript.
